# Repeated elicitation of the acoustic startle reflex leads to sensitisation in subsequent avoidance behaviour and induces fear conditioning

**DOI:** 10.1186/1471-2202-12-30

**Published:** 2011-04-13

**Authors:** Thomas Götz, Vincent M Janik

**Affiliations:** 1 Sea Mammal Research Unit, School of Biology, University of St Andrews, Fife KY16 8LB, UK

## Abstract

**Background:**

Autonomous reflexes enable animals to respond quickly to potential threats, prevent injury and mediate fight or flight responses. Intense acoustic stimuli with sudden onsets elicit a startle reflex while stimuli of similar intensity but with longer rise times only cause a cardiac defence response. In laboratory settings, habituation appears to affect all of these reflexes so that the response amplitude generally decreases with repeated exposure to the stimulus. The startle reflex has become a model system for the study of the neural basis of simple learning processes and emotional processing and is often used as a diagnostic tool in medical applications. However, previous studies did not allow animals to avoid the stimulus and the evolutionary function and long-term behavioural consequences of repeated startling remain speculative. In this study we investigate the follow-up behaviour associated with the startle reflex in wild-captured animals using an experimental setup that allows individuals to exhibit avoidance behaviour.

**Results:**

We present evidence that repeated elicitation of the acoustic startle reflex leads to rapid and pronounced sensitisation of sustained spatial avoidance behaviour in grey seals (*Halichoerus grypus*). Animals developed rapid flight responses, left the exposure pool and showed clear signs of fear conditioning. Once sensitised, seals even avoided a known food source that was close to the sound source. In contrast, animals exposed to non-startling (long rise time) stimuli of the same maximum sound pressure habituated and flight responses waned or were absent from the beginning. The startle threshold of grey seals expressed in units of sensation levels was comparable to thresholds reported for other mammals (93 dB).

**Conclusions:**

Our results demonstrate that the acoustic startle reflex plays a crucial role in mediating flight responses and strongly influences the motivational state of an animal beyond a short-term muscular response by mediating long-term avoidance. The reflex is therefore not only a measure of emotional state but also influences emotional processing. The biological function of the startle reflex is most likely associated with mediating rapid flight responses. The data indicate that repeated startling by anthropogenic noise sources might have severe effects on long-term behaviour. Future, studies are needed to investigate whether such effects can be associated with reduced individual fitness or even longevity of individuals.

## Background

The mammalian startle reflex is a fast motor response that is elicited if a tactile, vestibular or acoustic stimulus has a sudden onset and exceeds a certain intensity threshold [1] (see additional file 1: Movie 1 for a demonstration of a seal's startle response). The reflex involves a fast flexor muscle contraction (flinch) by which it can be distinguished from the orienting and defense reflexes [2]. The startle reflex can only be elicited by stimuli with certain acoustic parameters. For example, in rats, acoustic startle requires a stimulus to reach an intensity of 80-90 dB above the hearing threshold within about 15 ms of its onset [3]. The primary reflex is mediated by an oligo-synaptic reflex arc that involves the cochlea root nucleus, the caudal pontine reticular nucleus and spinal motoneurons [4]. Over the last four decades the startle reflex arc has become a prime model system for the study of the neuronal basis of sensory-motor integration [5], emotional processing [6] and the influence of genes on behaviour [7]. However, in spite of the extended research effort on the startle reflex, its evolutionary function remains unknown. Initially it has been argued that its function is to interrupt ongoing behaviour patterns [8] while more recent accounts suggest that it protects the organism from a sudden physical impact [1,9]. It has also been suggested that it facilitates a flight response [9], but there are no experimental studies on the connection between startling and fleeing in mammals. In previous studies, animals were not given the opportunity to spatially avoid the startle stimulus and remove themselves from sound exposure so that the relationship between startle and flight responses is not well understood. Furthermore, the startle reflex is commonly used as a measure of emotional processing [6] since the startle magnitude itself is often modified as result of conditioned fear [10] (fear to a conditioned stimulus (CS) formed by linking an initially neutral CS with an unconditioned aversive stimulus (US)). However, it is unknown whether startling noise itself can act as an US in a fear conditioning paradigm.

Information on sensitisation and habituation processes related to startle are only available for parameters that are directly associated with the reflex itself i.e. response latencies and startle amplitudes (strength of muscular flinch) [9]. The dual process theory of habituation [11] suggests that a stimulus should induce a habituating as well as a sensitisation component in the nervous system. Habituation constitutes a decreased response to repeated stimulation while response sensitisation represents the opposite process i.e. increased responsiveness as a result of repeated stimulation [11]. In the startle reflex, the magnitude of the startle itself (i.e. the strength of flexor muscle contraction) is subject to habituation while sensitisation is present in the shortening of the response latency to the startle stimulus [9,12]. However, no study has investigated whether repeated startling causes habituation or sensitisation processes in subsequent behaviour patterns like spatial avoidance or flight. In our study we address this question.

An understanding of long-term effects of the startle responses is important in the context of evolutionary and ecological questions and in the investigation of unexpected reactions to noise. The potential for exposure to repeated startling stimuli for wild animals has increased considerably through the introduction of anthropogenic noise. The effects of noise can range from habitat exclusion [13] to changes in the vocal parameters of communication sounds [14] to extreme behavioural responses leading to death as in mass strandings of beaked whales induced by military sonar [15-18]. Understanding the underlying mechanisms why mammals exhibit such responses is one of the highest priorities when trying to mitigate its effects [19]. In this study, we investigated the effects of repeated exposure to startle-eliciting stimuli on the occurrence of subsequent longer-term avoidance behaviour and fear conditioning.

## Results

### Experiment 1

**Figure 1:**
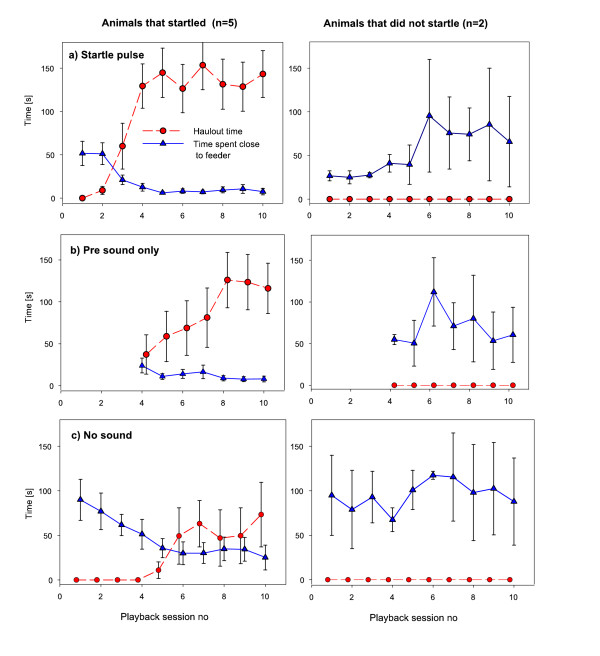
**Behavioural responses of seals (n = 7) to the three treatments in experiment 1**. Responses of seals that startled (left column) and those that did not startle (right column) to the treatments (a) startle pulse (with pre-sound), (b) pre-sound only, (c) no sound control. The response variables haulout time and time spent close to feeder are shown as mean +/- standard error. Note that the "pre-sound only" treatment was only used from session number 4 onwards.

In the first experiment, we exposed seven captive grey seals (*Halichoerus grypus*) to underwater noise pulses of 170 dB re 1 μPa (rms) while they were retrieving a fish from an underwater feeder. The feeder ensured that the animals were highly motivated to stay close to the loudspeaker since this was the location where they could retrieve fish. These startle pulses were 200 ms long with rise and fall times of 5 ms and exceeded the animal's hearing threshold by approximately 100 dB. The startle sound was always paired with a weaker (125-130 dB re 1 μPa), non-startling pre-sound (1.2 s duration, 100 ms rise and fall times) played 2 s before the startle stimulus to investigate whether the startle pulse can act as an unconditioned stimulus and the pre-sound as the conditioned stimulus in a fear conditioning paradigm. This lead time of 2 s was chosen to prevent pre-pulse inhibition of the startle response which is the phenomenon of a reduced startle magnitude if a non-startling sound precedes the startle pulse by 30-500 ms [5]. In the first experiment, five of seven seals showed clear signs of a startle response (flinches) while two did not. The animals that startled included 1 juvenile male, 3 juvenile females and 1 mature female. All animals that startled also showed a distinct sensitisation in subsequent avoidance behaviour, culminating in sustained avoidance of the exposure site (Figure 1a-c, left column and 2, see additional file 2: Movie 2 for a demonstration of the change in the seal's reactions over three consecutive playback sessions). We use the term 'subsequent avoidance behaviour' to refer to avoidance behaviours which followed sound exposure. Avoidance behaviour was quantified by a variety of response variables including time spent close to feeding station (underwater), time spent on land (haulout time), occurrence of flight behaviour followed by a jump out of the pool and prevention of fish retrieval. Sensitisation refers to 'response sensitisation' i.e. the fact that repeated stimulus presentation leads to increased responsiveness [11]. The sensitisation process in subsequent avoidance behaviour becomes obvious by the decreasing time the animals spent close to the feeding station and the rapid increase in the time they hauled out on land after several playback sessions (1 a).

A general linear model (GLM) was calculated in JMP 4 (SAS) to investigate the response variables 'time spent close to feeder' and 'haulout time' in further detail (Table 1). The model was calculated over the data set for the 5 animals that startled in experiment 1. Playback session number was included as an ordinal variable (playback session 1-10) while 'treatment', 'individual' and the interaction term 'treatment × individual' were included as categorical variables (factors). "Treatment" included three levels ("startle pulse", "pre-sound only", "no sound control") while "individual" included 5 levels i.e. the identity of the five seals. The model for the time spent close to the feeding station for the animals that sensitised was highly significant explaining 60% of the variance (GLM, F_23, 111 _= 9.12, p < 0. 0001, r^2^_adj _= 0.60). The factor with the strongest influence, determined by the F-value (table 1), was treatment (i.e. startle pulse, no sound or the pre sound only) followed by playback session number (ranging from 1 and 10). The interaction term of individual and treatment was not significant showing that all individuals responded in a similar way to the startling sound. The significant influence of playback session number indicates that the behaviour of each animal changed over the course of the experiment. In conjunction with the graphical evidence from (Figure 1a) this demonstrates that animals sensitised to exposure to the startle pulse. The model for haulout time was also highly significant (GLM, F_23, 111 _= 8.27, p < 0. 0001, r^2^_adj _= 0.56) and showed that treatment was by far the most important factor (determined by F-value, Table 1). The second most important factor was playback session number which showed that animals changed haulout behaviour over time. In all playbacks from session 3 the animals spent most time hauled out on land rarely entering the pool (Figure 1a). In later playback sessions, these responses were also caused by the pre-sound alone indicating that the pre-sound had acquired aversive properties as found in the conditioned stimulus in fear conditioning paradigms [10] (Figure 1b). Furthermore, from playback sessions 6 onwards avoidance of sound exposure turned into a more generic place avoidance which was exhibited even during the 'no sound control' treatment (see Figure 1c, e.g. increase in haulout time).

**Table 1:** General Linear Model (GLM) for the continuous response variables in experiment 1

	Time close		Time hauled out	
**Covariates**	**p**	**F**	**p**	**F**

Treatment	< 0.0001	29.8013	< 0.0001	32.9629
Individual	< 0.0001	8.2706	0.0288	2.8116
Playback session	< 0.0001	8.9398	< 0.0001	9.6711
Treatment × Individual	0.0534	1.995	0.0465	2.0528

Results of the GLM for the time spent close to the feeder and the time hauled out calculated over 5 animals that showed clear signs of startle reflex elicitation in experiment 1. The term 'treatment' refers to the sound exposure treatment, i.e. exposure to the startle pulse, the pre-sound only or the no sound control.

**Figure 2:**
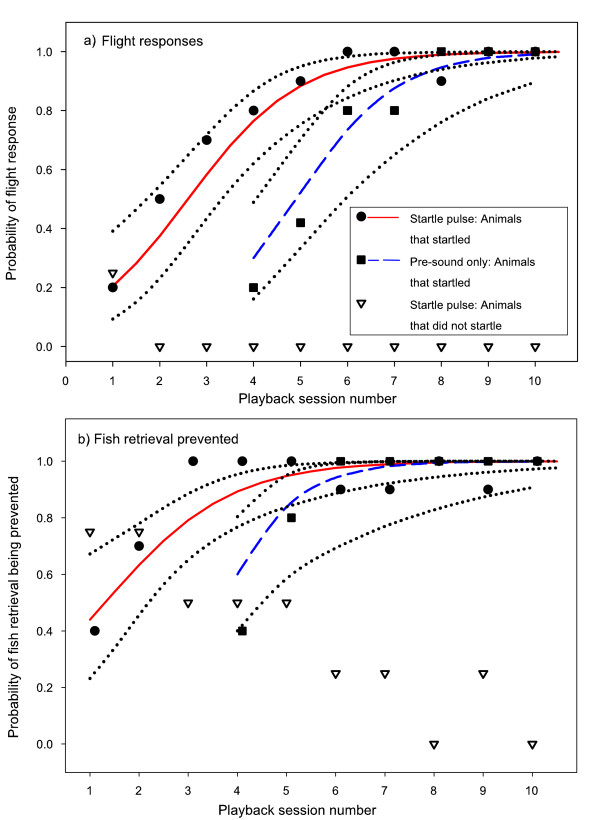
**Sensitisation of flight responses and interruption of foraging behaviour in response to the startle pulse and pre-sound**. Likelihood of sound presentations being followed by (a) an immediate fast flight response and (b) an instant interruption of foraging behaviour. Curves represent predicted values and their 5/95% confidence intervals derived from the logistic regression model fitted to the binomial raw data. Symbols represent the observed ratio of events averaged for each playback session. In animals that startled, exposure to the startle pulse caused marked sensitisation meaning that flight responses and the prevention of fish retrieval increased dramatically. In later playback sessions, the pre-sound alone caused a similar effect with respect to flight and interruption of foraging behaviour. Animals that did not startle habituated (inverted triangles).

In seals that startled, the sound pulse also prevented fish retrieval and increasingly caused an immediate rapid flight response which was followed by an erratic jump out of the pool indicating sensitisation to the startle pulse (Figure 2, additional file 2). After several pairings the pre-sound caused similar responses and reliably induced flight responses and prevented fish retrieval from the 6th playback session onwards. In contrast, seals that did not startle did not show sensitisation in any of the parameters but fish retrieval was less likely to be prevented in later playback sessions (Figure 1 a-c, right column). In the two seals that did not startle, the percentage of prevented fish retrieval decreased continuously indicating that animals habituated to sound exposure. The "pre-sound only" treatment did not interrupt foraging behaviour in animals that did not show signs of startle reflex elicitation. Flight responses never occurred during the no sound control and all seals were successful in retrieving the fish during all control sessions.

The likelihood of sound exposure causing a flight response was modelled for animals that startled using a logistic regression framework (Table 2). This was done to test for response sensitisation and model the change in the likelihood of aversive responses as a result of repeated exposure. The dependant variable (y) was given by the occurrence (1) or absence (0) of the respective event for each playback bout and each individual. The independent variables were given by the number of playback bouts for the two treatments (startle pulse and pre-sound). The final model was based on the combined binomial data for both treatments and included the single term 'playback bout by treatment' as independent variable. The model was based on 135 data points (startle pulse: 10 playback sessions with 2 bouts per session and 5 individuals, pre sound: 7 playback sessions with 5 individuals). The regression model for exposure to the startle pulse showed a sharp increase in the likelihood of flight responses after a few playback sessions (Figure 2, Table 2). The regression for the pre-sound only treatment showed a similar but delayed trend indicating that classical conditioning had formed a link between the pre-sound and the startle pulse after several more pairings (Figure 2, Table 2). The odds ratio estimates revealed that with each additional playback session the occurrence of a flight response became 2.3 and 2.6 times more likely as in the previous session for the startle pulse and pre-sound respectively (Table 2).

**Table 2:** Parameter estimates for the logistic regression models for occurrence of flight responses and interruption of foraging behaviour in animals that startled in experiment 1

	Flight responses				
	Chi-square	B (log. reg.coeff.)	Standard Error	p-value	**Odds ratio (e**^**B**^)
Startle pulse	23.05	0.424	0.088	< 0.001	1.53 (playback bout)
					2.34 (playback session)
Pre-sound only	15.91	0.934	0.234	< 0.001	2.55 (bout and session)
Intercept	10.97	-1.782	0.538	0.001	0.168
	**Fish retrieval prevented**				

Startle pulse	12.69	0.393	0.110	< 0.001	1.49 (playback bout)
					2.20 (playback session)
Pre-sound only	7.62	1.139	0.413	0.006	3.13 (bout and session)
Intercept	1.23	-0.634	0.571	0.267	0.530

Parameter estimates for the logistic regression models fitted to the data from experiment 1 (flight responses and fish retrieval in animals that startled). The odds ratio gives the increase in the odds of the respective event occurring with each additional playback bout and playback session.

The likelihood of food retrieval being interrupted showed an inverse pattern for the seals that showed signs of startle compared to those that did not. A sharp increase in the probability of foraging behaviour being interrupted was seen in animals that startled (Figure 2, Table 2). The model for the "pre-sound only" treatment for the animals that startled showed a similar but delayed increase in the likelihood of interruption of foraging (Figure 2, Table 2). This is also reflected by the odds ratios which showed that prevention of foraging behaviour became 2.2 (startle) and 3.1 (pre-sound) times more likely with each consecutive playback session

In summary, animals that startled developed an increasing reluctance to approach the feeding station and also exhibited behavioural responses generally associated with anxiety [20]. Such anxiety-related behaviour patterns include 'inhibition of ongoing behaviour' (interruption of foraging in Figure 2), avoidance of the source of danger (time spent close to feeder and haulout behaviour, Figure 1a & 1b) and 'scanning'. The latter was commonly observed when animals were hauled out on land with only the eyes in the water performing frequent erratic head turns (additional file 3) but it was not further quantified here.

### Experiment 2: Startle thresholds

Since we only observed distinctive startle responses (flinches) in some of the seals but not in all of them we conducted a second experiment using a step-wise procedure to determine the startle threshold for all animals (additional file1: Movie 1 shows an animal positioning in front of the feeder while being exposed to a pure tone stimulus of a certain sound pressure level). This second experiment revealed that all animals that startled and sensitised in the previous experiment had startle thresholds (50% response thresholds) between 155 and 160 dB re 1 μPa with a mean value across all 5 animals of 159 dB re 1 μPa. The mean value of 159 dB re 1 μPa reflects a sensation level of approximately 93 dB above the hearing threshold (see Table 3). We were unable to determine a startle threshold for the two animals that habituated in the first experiment as even the maximum level tested (180 dB re 1 μPa) did not elicit a startle response.

**Table 3:** Startle thresholds for various mammalian species expressed in units of sensation levels

Species	Startle threshold (pure tones): sound pressure level Underwater: re 1 μPa In air: re 20 1 μPa	Hearing threshold	Hearing threshold (average across studies)	Sensation level (dB re hearing threshold)
Human *Homo sapiens* (in air)	92 dB re 20 μPa; data taken from [42] (1 kHz) rise time: 5 ms (extrapolated)	dB (A) weighting	0 dB re 20 μPa	92 dB*
Rat *Rattus norvegicus* (in air)	Mean ranging from 85 - 95 dB re 20 μPa [9] (between 7 and 40 kHz) rise time: 5 ms	Mean ranging from 0 to 8 dB re 20 μPa (between 7 and 40 kHz) [9]	not used	87 dB**
Mouse *Mus musculus* (in air)	89 dB re 20 μPa (hybrid of strains) (5 kHz) [26] rise time: 0 ms	15 dB re 20 μPa [43] (house mouse)	only one study included	74 dB*
Grey seal *Halichoerus grypus* (underwater)	155-160 dB re 1 μ Pa mean: 159 dB (1 kHz, this study) rise time: 5 ms	76.6 dB re 1 μ Pa [44] 67 dB re 1 μ Pa [45] 54/56 dB re 1 μ Pa[46] (harbour seal, *Phoca vitulina*) data; behavioural audiogram for grey seals not available)	66 dB re 1 μ Pa (1 kHz, extrapolated)	93 dB*

* value derived from data in paper

** value directly reported in original publication

Table 3: Mammalian startle thresholds expressed in units of sensation levels. The data show that startle thresholds expressed in units of sensation level (level in dB above hearing threshold) are relatively uniform among mammals (if rise times of about 5 ms are used)

### Experiment 3: Is it the startle reflex that causes sensitisation?

**Figure 3:**
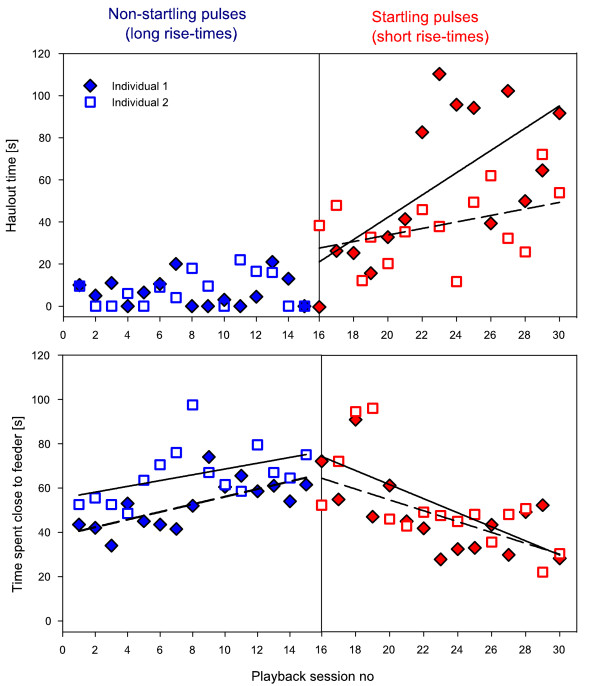
**Habituation and sensitisation process in response to the non-startling and startling sound pulses (experiment 3)**. Habituation and sensitisation of behavioural avoidance as measured by mean haulout time and time spent close to feeder for two seals tested consecutively with non-startling (long rise time) and startle-eliciting (short rise time) stimuli. Lines show separate linear regression through the data points for each individual. These seals were not used in any of the other experiments.

In a third experiment, we tested whether elicitation of the startle reflex arc is required to induce flight responses and subsequent avoidance behaviour or if any sound with a high sound pressure level is sufficient to elicit the same type of response. We first exposed two naive seals to playbacks of longer non-startling signals of acoustic energy and maximum sound pressure level equal to that of the startle stimuli from the first experiment (see additional file 4). However, these stimuli had longer rise times and were therefore unable to elicit the startle reflex [2,3]. Then we exposed them to the startle-eliciting sounds. The animals showed habituation to sounds of equal energy as the startle stimulus but with a longer rise time of 100 ms, whereas they sensitised in the subsequent test using the startle-eliciting stimulus with a 5 ms rise time (Figure 3). Linear regressions were calculated for each individual. Regressions revealed that repeated exposure to short rise time pulses resulted in increased haulout behaviour (Individual 1:p = 0.007, r^2 ^= 0.44, Individual 2:p = 0.04, r^2 ^= 0.29). In contrast, haulout behaviour only occurred rarely when animals were exposed to long rise time, non-startling sound pulses. Similarly, exposure to short rise time (non-startling) pulses resulted in a reduction of time spent close to the feeder over the course of the 15 playback sessions (Ind 1: r^2 ^= 0.39, p = 0.0012, Ind 2: r ^2 ^= 0.47, 0.0048). Exposure to long rise times showed the opposite pattern with one individual spending significantly more time close to the feeder in later playback sessions (Ind 1: r ^2 ^= 0.50, p = 0.0034, Ind 2: r ^2 ^= 0.21, p = 0.08). Thus, eliciting the startle reflex was required for causing increased responsiveness in later playback sessions and therefore inducing sensitisation of the avoidance responses.

## Discussion

This is the first demonstration that the startle reflex leads to sensitisation of an extreme avoidance response which constitutes a rare example of a sustained sensitisation process tied to a simple reflex. Sensitisation in the sense of an increased responsiveness to the stimulus was found in a variety of response variables i.e. increased haulout time and reduced time spent close to the feeder in later playback sessions. Similarly, the likelihood of flight responses occurring increased rapidly in later playback sessions which is another example of response sensitisation. Treatment with long rise time stimuli on the other hand led to a waning of avoidance responses indicating habituation in experiment 3. This shows that it was the startle reflex and not the defense reflex that caused sensitisation of flight behaviour. Long rise time, high intensity stimuli can elicit the defence reflex [2,21], which has been interpreted as part of the fight and flight reaction of animals [21]. However, using such stimuli, the animals showed a quick habituation (decreased responsiveness) of avoidance behaviour and decreasing frequency of flight responses. These results are also consistent with our earlier study that tested grey seal avoidance behaviour in response to non-startling, longer duration sound types in which seals were found to habituate rapidly i.e. flight responses waned, animals spent increasing amounts of time close to the feeder and never hauled out during the experiment [22]. Three of the test subjects that sensitised in experiment 1 where also used in our previous study in which they habituated to all stimuli i.e. avoidance behaviour waned. Thus, while the defence reflex might be involved in initial flight responses, in our study only elicitation of the startle reflex resulted in sensitisation of avoidance responses and increased the likelihood of flight responses.

A comparison of the startle threshold from this study with previous studies showed that the startle threshold expressed in units of sensation levels (dB above hearing threshold) is similar to the sensation level required to induce startle in rats [23] and humans [24] (Figure 3 and Table 3). Thus, the startle threshold may be fairly universal and conserved among mammals in spite of specific adaptations to aquatic hearing in seals (Table 3). The two seals that did not show sensitisation in subsequent avoidance behaviour in experiment 1 also never showed an observable startle response, not even at the highest tested received level of 180 dB re 1 μPa (experiment 2) This suggests that elicitation of the startle reflex was necessary to evoke sensitisation of avoidance responses in these animals. We suspect that the two non-startling animals had impaired hearing since they were among the oldest animals tested and because in mice the sound pressure level required to elicit a startle response increases with hearing loss [25]. While the exact threshold in mammals depends not only on the received level of the sound but also on stimulus duration and rise time [26], the sensation level value typically lies at about 90 dB above the hearing threshold if rise times of about 5 ms are used and the duration is kept constant (Table 3). This sensation level remains similar to the original level even when age-related hearing loss sets in (i.e. absolute startle thresholds rise with increasing hearing loss [25]). One study on mice [26] found a lower startle threshold (Table 3) but used stimuli with an almost instantaneous rise time which is known to lower the required intensity threshold for a startle response. The majority of the animals tested in our study were females. Even though the only male that we tested also sensitised, it would be interesting to explore sex differences in these responses in more detail. In mice, males have higher startle magnitudes and more pronounced long-term habituation of startle magnitudes than females [27]. Gonadal hormones such as estradiol- and dihydrotestosterone on the other hand can cause a decrease in startle magnitude in rats with gonadectomy [28]. However, it is important to note that it is unclear whether the magnitude of the startle reflex (strength of muscular contraction) is in any way related to the aversive follow-up response (avoidance, flight) observed in our experiments. Furthermore, startle modulation as a result of sex or hormonal differences is unlikely to explain the lack of observable startle responses found in two of the females that habituated.

The behavioural responses observed in experiment 1 and 3 are remarkably similar to those observed in studies that involved electric stimulation of the brain. Repeated electrical stimulation of the amygdala or the defence circuitry in the superior colliculus leads to long-term sensitisation resulting in anxiogenic-type consequences and pronounced flight response to subsequent stressors [29,30]. Similarly, stimulation of the acoustic pathway in the inferior colliculus at increasing intensities first leads to freezing and then ultimately to escape behaviour [31] and sensitisation [32]. Although the primary startle pathway is thought to be mediated by the cochlea nucleus which projects into the pontine reticular formation [4,5], the latter structure also receives indirect acoustic input from the inferior colliculus [33]. Furthermore, previous studies have shown that the magnitude of the startle reflex can be increased by fear-inducing experiences [6] and startle has long been used as an indicator of fear [6] and emotional state [6]. Our data showed that a startle stimulus can act as an unconditioned stimulus in a fear conditioning paradigm, as also suggested by an ethically questionable experiment on one human baby [34]. Thus, the startle reflex is not only influenced by emotional state [6] but repeated exposure to startling stimuli appears to cause fear. This indicates the presence of an afferent input from the startle pathway to brain areas related to emotional processing such as the amygdala and shows that the mammalian startle reflex evolved most likely in the context of general predator avoidance. Interestingly, the projection from the startle pathway to the amygdala [35] and its effects has received little research attention while the efferent connection from the amygdala to the startle pathway is of great significance in major research efforts using fear-potentiated startle as an indicator of fear conditioning through other stimuli and as a measure of emotional valence of such stimuli [6,36,37].

The startle reflex is commonly used as a measure for emotional processing in studies on human anxiety disorders [38]. Patients with panic disorder, post-traumatic-stress disorder (PTSD) or obsessive compulsory disorder (OCD) generally show elevated baseline startle magnitudes [38]. Our study indicates that repeated startling influences emotional processing. Thus, the potential role of repeated startle elicitation in the development of post-traumatic stress disorder should be considered. The main behavioural categories thought to characterize post-traumatic stress disorder (PTSD) in animal models are "conditioned behaviours" (i.e. fear conditioning) and "sensitised behaviours" (e.g. hypervigilance) [39]. In our study, we found evidence for both "conditioned" (fear conditioning) and "sensitised behaviour" (increasing flight responses, reluctance to approach feeder) as a result of exposure to repeated startling stimuli. While the severity of the behaviour patterns observed in this study is probably less strong than in the PTSD model, our data show that long-term exposure of humans to pulsed noise should be critically evaluated.

We think it is likely that the original function of the startle reflex is associated with increasing an animal's propensity for flight as required in a predator avoidance scenario [9]. If the biological function of the startle reflex was primarily associated with injury prevention through increased muscle tonus [1] we would have expected an absence of flight and avoidance responses as a result of startle elicitation. Many startling sounds indicate serious threats caused by predators. These include sounds of breaking tree branches, falling rocks or the sudden impact noise of a predator attacking a conspecific. A sensitisation to startle sounds as observed in our study would be beneficial not only by enabling a rapid predator avoidance response but also by preventing an animal from moving into an area with an increased threat level where startling sounds are encountered repeatedly. Interestingly, animals may also exploit the startle reflex to manipulate conspecific, prey or predator behaviour. For instance, cod were found to produce potentially startle eliciting clicks before prey capture attempts by seals [40]. Bottlenose dolphins produce high-intensity jaw pops as a threat display during courtship which could potentially startle conspecifics [41]. Future research will be needed to address the question whether basic reflexes like the startle have shaped the evolution of communication signals.

In contrast to most neuro-physiological studies, the animals we tested here were captured from a wild population, where they had spent time in their natural habitat prior to the experiments. They also belonged to a taxon that is not closely related to any of the standard model systems. Our reason to choose the grey seal as a test species was partly a concern over observed detrimental responses of marine mammals to noise pollution. There are many anthropogenic noise sources in use that can cause startle responses. Gun shots and some industrial noise are examples in air. However, most pulsed noise caused by human activity can be found in the marine environment such as in underwater explosions, pile-driving activities, acoustic deterrent devices, sonar pulses and seismic air guns. Marine mammals have been found to abandon areas of high noise pollution [13] and even strand as an extreme behavioural avoidance response to military sonar [15-18]. While the role of the startle reflex in these reactions needs further study, it is notable that sonar sounds often have a very rapid onset and high source level. Our results showed that a simple oligo-synaptic reflex arc is responsible for extreme avoidance responses to sudden-onset, pulsed sounds. Thus, impact ratings of anthropogenic noise sources in air and in water should be re-evaluated and rise times of loud noise pulses should be increased to mitigate their effects on humans and animals alike.

## Conclusion

Acoustic stimulation of the startle reflex pathways leads to sensitisation of extreme avoidance behaviour and induces sustained flight response in mammals. Hence, sensitisation of longer-term follow-up behaviour can be caused by repeated stimulation of a simple oligo-synaptic reflex arc. Furthermore, startling stimuli are capable of inducing fear conditioning. This shows the startle may not only be a measure of emotion (as used in biomedical sciences) but might influence the emotional state of an animal itself. The primary function of the startle reflex circuitry therefore seems to be associated with predator avoidance behaviour through induction of rapid flight responses. Startle-eliciting noise pulses have the potential to cause severe effects on long-term behaviour, individual fitness and longevity of individuals in wild animal populations. For anthropogenic noise sources we could mitigate against such effects by increasing stimulus rise times. Repeated long-term exposure of humans to short rise time pulsed noise also may be problematic and acoustic startle should be considered as a potentially contributing factor in the context of post-traumatic stress disorder.

## Methods

Grey seals (*Halichoerus grypus*) were caught off the East coast of Scotland. All work described in this manuscript has been carried out under Home Office Licence Number 60/3303. Seven animals were tested in experiment 1, three adult females, three juvenile females and one juvenile male. The juveniles were just under one year of age. The experimental pool was circular with a 3 m diameter and 1.5 m of water depth. Sounds were played from a Panasonic SL-S120 CD player, using a Phonic Mar2 amplifier and a Lubell 9162 underwater loudspeaker for projection. Transducer calibration and sound field measurements were conducted using a B & K 8103 hydrophone, a B & K 2635 charge amplifier and the calibrated sound card of a laptop computer.

All data sets were tested for normality by using Kolmogorov-Smirnov tests prior to statistical testing and, if necessary, data were transformed by log_10 _(x). General Linear Models were calculated in JMP 4 (SAS) while linear regressions were fitted in Sigma Plot 8 (SPSS Inc.) The calculation of the GLM and the linear regressions also included tests for autocorrelation of the residuals and constant variance. Logistic regression models were calculated using the "Generalized Linear Model" platform in PASW 18 (SPSS Inc.). The distribution was specified as 'binomial' and the link function was specified as 'logit'. Data were arranged by playback bouts which were consecutively numbered based on their order in the experiment i.e. bout numbers for the startle pulse were 1-20 while bout numbers for the pre-sound were 1-7. The data were then combined into an overall model that contained 135 data points (20 startle bouts for each of the 5 individuals, 7 pre-sound bouts for each of the 5 individuals). The model was calculated on a "playback bout" scale since each additional bout constituted a new event of sound presentation that is likely to change the animals' behaviour. However, the predicted values (obtained from the modelling output in PASW18) were plotted on the "playback session number" scale (Figure 2) since "playback session" represented the repeated, finite series of treatments used in the experiment. The final model only contained the nested term "bout number by treatment". The approach of including a single term "bout by treatment" is identical to including two covariates (startle bout number, pre-sound bout number) with respective missing values in the other covariate set to 0. This simplest form of the model also had the lowest AIC and included the maximum number of significant variables. To compare between the two treatments, odd ratios are given for both, playback bout and playback session number.

### Experiment 1

The seal was attracted to a feeding tube by lowering a metal cup that contained a fish in it. The playbacks started when the tip of the nose of the animal was within 40 cm of the end of the tube (1 m from the speaker). The startle pulse was a band-limited noise pulse with a peak frequency of 950 Hz spanning approximately 2 octaves. Ambient noise levels in the test pool were published elsewhere [22]. The sound field in the pool was measured at 0.8 and 1.2 m depth and received levels ranged from 170-174 dB re 1 μPa. The received level at the position of the animals head at the start of the playback was 170 dB re 1 μPa. The pre-sound was a tone of 1.2 s duration which was frequency modulated at a modulation rate of 3 Hz between 700 and 1300 Hz. Playback sessions were conducted over three consecutive days always separated by at least 20 min and an additional 3 hour break after two consecutive sessions. Each seal took part in 10 playback sessions. A playback session consisted of 3 or 4 bouts of 3 min observation periods which were separated by a 5 min pause: a) no sound treatment, b) two bouts with the startle pulses preceded by the pre-sound c) pre-sound only (after the 3rd playback session). In the startle bouts the seal heard the stimuli either once or twice depending on whether it left the pool or re-approached the feeder. From underwater video recordings we scored the occurrence of an initial startle reflex, any flight responses, the interruption of feeding, the distance of the animal from the feeding station and the time the animal spent on land. The animals had been trained to use the feeder but were not fed prior to the experiment in order to make them motivated to approach.

Two seconds after the playback started the cup was lowered and gave the seal access to the food. Reactions to playbacks were recorded with two video cameras, one above the pool and one underwater (see additional file 2: Movie 2 for pictures of the experimental setup). We scored behavioural responses from the video, including whether or not the animal startled (i.e. showed a visible flinch), whether it showed a fast acceleration away from the feeder (flight response), whether it successfully retrieved the fish, how long its head stayed within 1.5 m of the feeder, and how much time the animal spent outside the pool (haulout time).

### Experiment 2

Startle thresholds were measured separately after the initial experiment, using the same experimental setup. For startle threshold tests, we used a 1 kHz pure tone stimulus with a 200 ms duration and a 5 ms rise time. This sound was presented when the animal was stationary within 40 cm of the feeder. We presented nine intensity levels (received levels of 140-180 dB re 1 μPa in increments of 5 dB) in a pseudo-randomized order to each seal (1 min inter-stimulus interval). The highest level of 180 dB re 1 μPa was only played twice. This procedure was repeated 4 times with each seal. Playback session were recorded with an underwater camera and consecutively analysed for any sign of flexor muscle contraction during the playback. In order for a response to be considered a "startle" an animal had to exhibit at least a clear neck flinch but often whole body startle was observed. A seal had to startle in two out of four presentations of the same intensity level to be considered startling to that level (50% response threshold). Received levels were measured at positions where the seal's head was in the experiment and varied by ± 3 dB.

### Experiment 3

We used two naïve, female juvenile grey seals for the third experiment that compared reactions to startling and non startling sounds of the same maximum sound pressure level and the same acoustic energy. The startle-eliciting pulse was the same as in experiment 1. In contrast, the non-startling pulse was designed to have a longer rise time of 100 ms but the same acoustic energy and the same average root-mean-square (rms) sound pressure level within the centre section. The non-startling sound was 295 ms long with 100 ms rise and fall times and a flat 95 ms long central section. No pre-sounds were used but the experimental setup was otherwise identical to experiment 1. We first exposed each animal to 15 trials of the long rise time stimulus, followed by 15 sessions with the short rise time stimulus with both blocks being separated by 1 day.

## Authors' contributions

TG participated in conceiving and designing the study, carried out the experimental work, analysed the data and participated in drafting the manuscript. VMJ participated in conceiving and designing the study, raised the funding, coordinated the project and participated in drafting the manuscript. All authors read and approved the final manuscript.

## Supplementary Material

Additional file 1 **Startle threshold measurement in experiment 2**. The video shows the behaviour of a female, juvenile grey seal in experiment 2 (measuring the startle threshold). We exposed the animal to a pure-tone pulse when it was observed to float motionless in front of the feeder. In the video the animals shows a clear startle response (whole body flinch). Click here for file

Additional file 2 **Sensitisation process caused by repeated exposure to startling stimuli in experiment 1**. The video shows the sensitisation process of a female juvenile grey seal during the initial bouts of the first three playback sessions (labelled as 'playback') in experiment 1 (treatment: pre-sound + startle pulse). In the first playback session the seal only exhibits a startle but no lasting flight response. In the second playback session, (= after three playbacks) the pre-sound already begins to establish aversive properties but the animal only exhibits a flight responses on hearing the startle pulse. In the third playback session (= after 5 playbacks) the animal responds with a rapid jump out of the pool in response to the pre-sound. Click here for file

Additional file 3 **Seal exhibiting scanning behaviour while hauled out**. Seals that sensitised often exhibited head scanning behaviour towards the end of the experiment. For this, they typically stayed on land with just the front of the head submerged in the pool performing frequent head turns. Click here for file

Additional file 4 **Graph visualising the startling and non-startling stimuli used in experiment 1 & 3**. Envelope of the sound stimuli used in experiment 1 and 3. Both noise pulses differed in their rise times but had equal acoustic energy (grey area) and equal maximum sound pressure level (p-p and rms) during the flat centre section (marked by dashed lines). Click here for file

## References

[B1] Yeomans JS Li L Scott BW Frankland PW Tactile, acoustic and vestibular systems sum to elicit the startle reflex Neurosci Biobehav Rev 2002 12 1 11 10.1016/S0149-7634(01)00057-4 11835980

[B2] Graham FK Kimmel HD, van Olst EH, Orlebeke JF Distinguishing among orienting, defense, and startle reflexes The orienting reflex in humans 1979 Hillsdale, NJ: Lawrence Erlbaum Associates 137 167

[B3] Fleshler M Adequate acoustic stimulus for startle reaction in the rat J Comp Physiol Psychol 1965 12 200 207 10.1037/h0022318 5832345

[B4] Lee YL Lopez DE Meloni EG Davies MA A primary acoustic startle pathway: Obligatory role of cochlear root neurons and the nucleus reticularis pontis caudalis J Neurosc 1996 12 3775 3789 10.1523/JNEUROSCI.16-11-03775.1996 PMC6578836 8642420

[B5] Koch M The neurobiology of startle Prog Neurobiol 1999 12 107 128 10.1016/S0301-0082(98)00098-7 10463792

[B6] Lang PJ Davis M Emotion, motivation, and the brain: reflex foundations in animal and human research Prog Brain Res 2006 12 3 29 full_text 17015072 10.1016/S0079-6123(06)56001-7

[B7] Plappert CF Pilz PKD The acoustic startle response as an effective model for elucidating the effects of genes on the neural mechanism of behavior in mice Behav Brain Res 2001 12 183 188 10.1016/S0166-4328(01)00299-6 11682109

[B8] Landis C Hunt WA The startle pattern 1939 New York: Ferrar and Rinehart

[B9] Pilz PKD Schnitzler HU Habituation and sensitisation of the acoustic startle response in rats: amplitude, threshold, and latency measures Neurobiol Learn Mem 1996 12 67 79 10.1006/nlme.1996.0044 8661252

[B10] Brown JS Kalish HI Farber IE Conditioned fear as revealed by magnitude of startle response to an auditory stimulus J Exp Psychol 1951 12 317 328 10.1037/h0060166 14861383

[B11] Groves PM Thompson RF Habituation: A dual process theory Psychol Rev 1970 12 419 450 10.1037/h0029810 4319167

[B12] Ornitz EM Guthrie D Long-term habituation and sensitization of the acoustic startle response in the normal adult human Psychophysiology 1989 12 166 173 10.1111/j.1469-8986.1989.tb03149.x 2727218

[B13] Morton AB Symonds HK Displacement of *Orcinus orca *(L.) by high amplitude sound in British Columbia, Canada ICES J Mar Sci 2002 12 71 80 10.1006/jmsc.2001.1136

[B14] Brumm H Slabbekoorn H Acoustic communication in noise Advan Study Behav 2005 12 151 209 full_text

[B15] Frantzis A Does acoustic testing strand whales? Nature 1998 12 29 10.1038/32068 9510243

[B16] Jepson PD Arbello M Deaville R Patterson IA Castro P Baker JR Degollada E Ross HM Herraez P Pocknell AM Pocknell AM Rodriguez F Howie FE Espinosa A Reid RJ Jaber JR Martin V Cunningham AA Fernandez A Gas-bubble lesions in stranded cetaceans Nature 2003 12 575 576 10.1038/425575a 14534575

[B17] Nowacek DP Thorne LH Johnston DW Tyack PL Responses of cetaceans to anthropogenic noise Mam Rev 2007 12 81 115 10.1111/j.1365-2907.2007.00104.x

[B18] Weilgart LS The impacts of anthropogenic ocean noise on cetaceans and implications for management Can J Zool 2007 12 1091 1116 10.1139/Z07-101

[B19] Southall BL Bowles AE Ellison WT Finneran JJ Gentry RL Greene CR Kastak D Ketten DR Miller JH Nachtigall PE Richardson WJ Thomas JA Tyack PL Marine mammal noise exposure criteria: Initial scientific recommendations Aquat Mam 2008 12 411 521 10.1578/AM.33.4.2007.411

[B20] Belzung C Griebel G Measuring normal and pathological anxiety-like behaviour in mice: a review Behavioural Brain Research 2001 12 141 149 10.1016/S0166-4328(01)00291-1 11682105

[B21] Turpin G Schaefer F Boucsein W Effects of stimulus intensity, rise time, and duration on autonomic and behavioral responding: implications for the differentiation of orienting, startle, and defense responses Psychophysiology 1999 12 453 463 10.1111/1469-8986.3640453 10432794

[B22] Götz T Janik VM Aversiveness of sounds in phocid seals: psycho-physiological factors, learning processes and motivation J Exp Biol 2010 12 1536 1548 20400639 10.1242/jeb.035535

[B23] Pilz PKD Schnitzler HU Menne D Acoustic startle threshold of the albino rat (*Rattus norvegicus*) J Comp Psychol 1987 12 67 72 10.1037/0735-7036.101.1.67 3568609

[B24] Blumenthal TD Berg WK Stimulus rise time, intensity, and bandwidth effects on acoustic startle amplitude and probability Psychophysiology 1986 12 635 641 10.1111/j.1469-8986.1986.tb00682.x 3823338

[B25] Ouagazzal AM Reiss D Romand R Effects of age-related hearing loss on startle reflex and prepulse inhibition in mice on pure and mixed C57BL and 129 genetic background Behav Brain Res 2006 12 307 315 10.1016/j.bbr.2006.05.018 16814879

[B26] Stoddard CW Noonan J Martin-Iverson MT Stimulus quality affects expression of the acoustic startle response and prepulse inhibition in mice Behav Neurosci 2008 12 516 526 10.1037/0735-7044.122.3.516 18513122

[B27] Plappert CF Rodenbücher AM Pilz PKD Effects of sex and estrous cycle on modulation of the acoustic startle response in mice Physiol Behav 12 585 10.1016/j.physbeh.2005.02.004 15811394

[B28] Turvin JC Messer JWS Kritzer MF On again, off again effects of gonadectomy on the acoustic startle reflex in adult male rats Physiol Behav 12 473 10.1016/j.physbeh.2006.10.010 17169383 PMC2517218

[B29] Helfer V Deransart C Marescaux C Depaulis A Amygdala kindling in the rat: anxiogenic-like consequences Neuroscience 1996 12 971 978 10.1016/0306-4522(96)00081-4 8809816

[B30] King SM Escape-related behaviours in an unstable, elevated and exposed environment II. Long-term sensitization after repetitive electrical stimulation of the rodent midbrain defence system Behav Brain Res 1999 12 127 142 10.1016/S0166-4328(98)00061-8 10210529

[B31] Lamprea MR Cardenas FP Vianna DM Castilho VM Cruz-Morales SE Brandao ML The distribution of fos immunoreactivity in rat brain following freezing and escape responses elicited by electrical stimulation of the inferior colliculus Brain Res 2002 12 186 194 10.1016/S0006-8993(02)03036-6 12231243

[B32] Pandossio JE Molina VA Brandao ML Prior electrical stimulation of the inferior colliculus sensitizes rats to the stress of the elevated plus-maze test Behav Brain Res 2000 12 19 25 10.1016/S0166-4328(99)00154-0 10699654

[B33] Hori A Yashuara A Naito H Yashuara M Blink reflex elicited by auditory stimulation in the rabbit J Neurol Sci 1986 12 49 59 10.1016/0022-510X(86)90141-3 3783188

[B34] Watson JB Rayner R Conditioned emotional reactions J Exp Psychol 1920 12 1 14 10.1037/h0069608 10743250

[B35] Ebert U Koch M Acoustic startle-evoked potentials in the rat amygdala: effect of kindling Physiol Behav 1997 12 557 562 10.1016/S0031-9384(97)00018-8 9272664

[B36] Koch M Schnitzler HU The acoustic startle response in rats - circuits mediating evocation, inhibition and potentiation Behav Brain Res 1997 12 35 49 10.1016/S0166-4328(97)02296-1 9475613

[B37] Maren S Neurobiology of Pavlovian fear conditioning Ann Rev Neurosci 2001 12 897 931 10.1146/annurev.neuro.24.1.897 11520922

[B38] Grillon C Startle Reactivity and Anxiety Disorders: Aversive Conditioning, Context, and Neurobiology Biol Psychiat 2002 12 958 975 10.1016/S0006-3223(02)01665-7 12437937

[B39] Siegmund A Wotjak CT A mouse model of posttraumatic stress disorder that distinguishes between conditioned and sensitised fear J Psychiatr Res 2007 12 848 10.1016/j.jpsychires.2006.07.017 17027033

[B40] Vester HI Folkow LP Blix AS Click sounds produced by cod (*Gadhus morhua*) Journal of the Acoustical Society of America 2004 12 916 919 10.1121/1.1639106 15000203

[B41] Connor RC Smolker RA 'Pop' goes the dolphin: A vocalization male bottlenose dolphins produce during consortships Behaviour 1996 12 643 662 10.1163/156853996X00404

[B42] Berg KM Elicitation of acoustic startle in the human PhD thesis 1973 University of Wisconsin

[B43] Heffner H Masterton B Hearing in glires-domestic rabbit, cotton rat, feral house mouse, and kangaroo rat J Acoust Soc Am 1980 12 1584 1599 10.1121/1.385213

[B44] Kastak D Schusterman RJ Low-frequency amphibious hearing in pinnipeds: methods, measurements, noise and ecology J Acoust Soc Am 1998 12 2216 2228 10.1121/1.421367 9566340

[B45] Terhune JM Detection thresholds of a harbor seal to repeated underwater high-frequency, short-duration sinusoidal pulses Can J Zool 1988 12 1578 1582 10.1139/z88-230

[B46] Kastelein RA Wensveen PJ Hoek L Verboom WC Terhune JM: Underwater detection of tonal signals between 0.125 and 100 kHz by harbor seals (*Phoca vitulina*) J Acoust Soc Am 2009 12 1222 1229 10.1121/1.3050283 19206895

