# Supplementary figures and images for: Repeated elicitation of the acoustic startle reflex leads to sensitisation in subsequent avoidance behaviour and induces fear conditioning
Source: BMC Neurosci. 2011 Apr 13;12:30. doi: 10.1186/1471-2202-12-30 (PMC3101131; doi:10.1186/1471-2202-12-30)

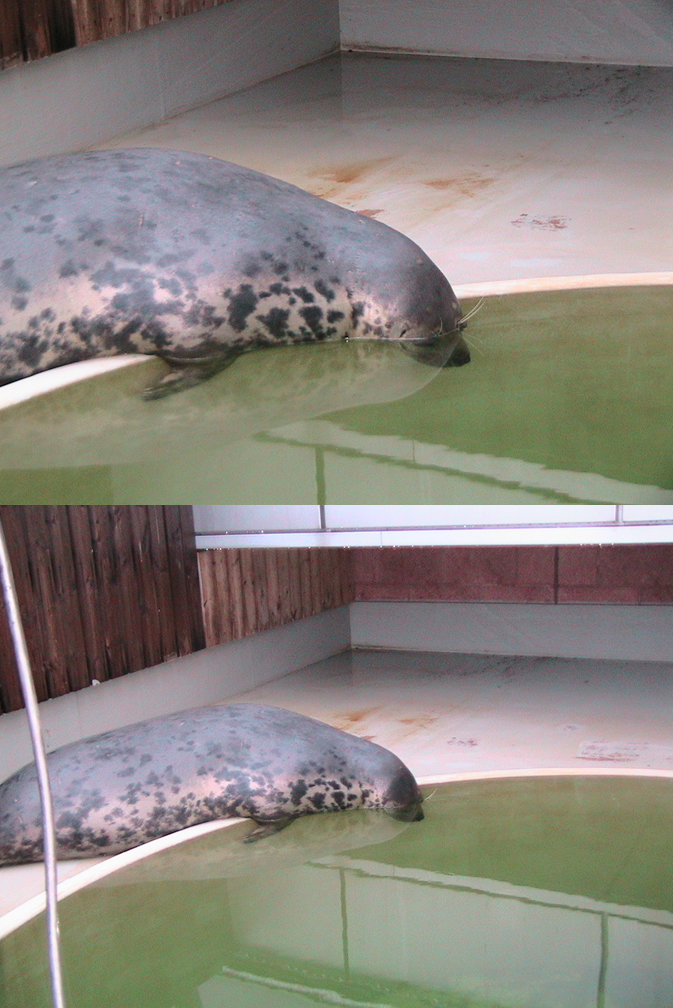

Supplement: Additional file 3 — Seal exhibiting scanning behaviour while hauled out. Seals that sensitised often exhibited head scanning behaviour towards the end of the experiment. For this, they typically stayed on land with just the front of the head submerged in the pool performing frequent head turns. [file 1471-2202-12-30-S3.TIFF]

Short rise-time, startling pulse (experiment 1 & 3)

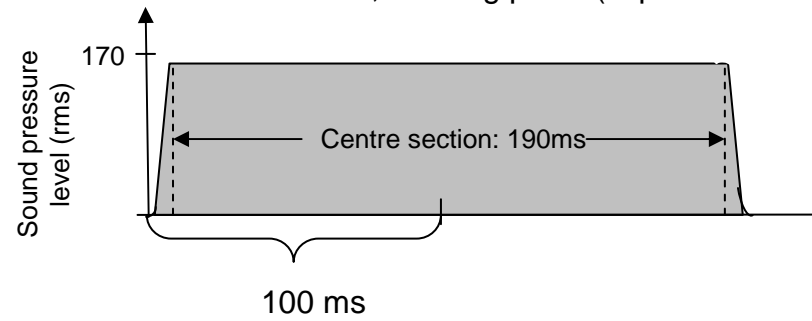

Long rise-time, non-startling pulse (experiment. 3)

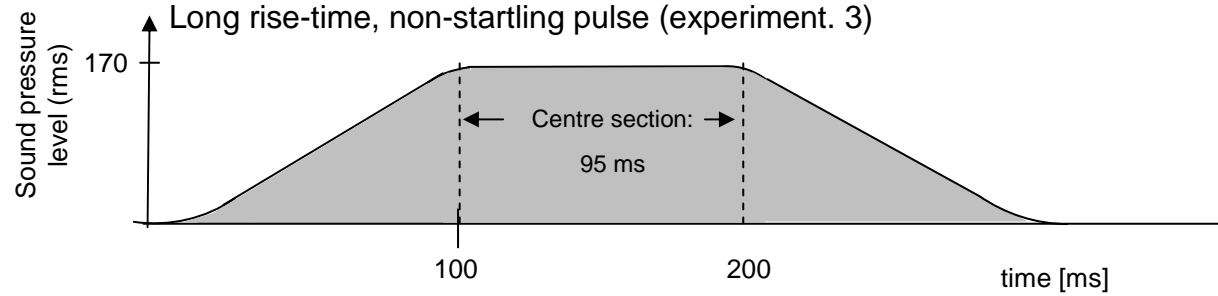

Supplement: Additional file 4 — Graph visualising the startling and non-startling stimuli used in experiment 1 & 3. Envelope of the sound stimuli used in experiment 1 and 3. Both noise pulses differed in their rise times but had equal acoustic energy (grey area) and equal maximum sound pressure level (p-p and rms) during the flat centre section (marked by dashed lines). [file 1471-2202-12-30-S4.PDF]
